# Protecting Against Unintended Risk From Student Quality Improvement Projects in Health Care

**DOI:** 10.63116/ahisp-25-009

**Published:** 2026-04-17

**Authors:** Ianthe Bryant-Gawthrop

**Affiliations:** Department of Educational Studies and Department of Biological Sciences, Purdue University

**Keywords:** Quality improvement, HIPAA, student projects, risk mitigation, privacy, data security, IRB

## Abstract

Quality improvement (QI) project design is an essential characteristic of degree programs for future health care leaders. The use of evidence-based practices strengthens patient safety and organizational efficiency. The curriculum for advanced degree programs emphasizes and requires a QI project for each student, often allowing the activity to occur at the student’s place of employment, thus creating a dual responsibility (student-employee). In the past two decades, institutional review boards (IRBs) and practitioners have struggled with incorporating uniformity in the review of research versus QI design. Despite the efforts of experts in ethics and calls to develop formal processes, consistency issues are still a challenge in traditional IRB review models. The process to mandate the IRB as the sole decision-maker for QI projects may lead to overregulation and bottlenecks in the institutional workflow. In this report, the author attempts to reconcile the causes of overregulation and also draw attention to potentially unrealized risks to patient privacy and protections. The proposed effort aims to (1) proactively establish when governance models are necessary for the population of student-employees conducting QI projects, and (2) promote interaction and cooperation when addressing ethical and regulatory standards outside of the IRB to reduce overregulation for student-employee QI projects. The author also describes a novel heatmap for risk identification and introduces the 5Circle Student Employee Venn for Quality Improvement model as a proposed mechanism to help institutions design more appropriate governance structures for student-employee QI projects. Finally, the author suggests ways by which institutions can increase efficiency and reduce overregulation of QI.

## Background

The quality improvement (QI) process introduces organized efficiency to individual systems.^1–3^ Health care organizations (eg, hospitals, clinics, and pharmacies) often use evidence-based practices to optimize processes and deliver the highest standards of care to their patients.^3–7^ The US Health Resources and Services Administration defines *quality improvement* as, “An organizational philosophy that seeks to meet client needs and expectations with the minimum of effort or rework or waste, by using a structured process that selectively identifies and improves all aspects of care and service on an ongoing basis.”^8^

Topics of QI inquiry may include passive or active studies, such as medical chart review of patient outcomes, employee and patient education, or intentional, responsive changes in standards of care.^3^^,^^4^^,^^7^ Applying evidence-based methodologies can aid institutions in advancing practices beyond the status quo. QI is considered critical for adopting innovations and for maximizing the use of available budgets and workforce efficiency.^3^^,^^5^^,^^6^^,^^9^^,^^10^ Furthermore, publication of a QI effort demonstrates awareness of implementation and success measures that reflect overcoming challenges and barriers to improving operations.^11–14^

The complexity of a QI project increases when data inputs and outputs originate from an electronic medical record or contain other protected health information (PHI) subject to the Health Insurance Portability and Accountability Act (HIPAA) Privacy Rule.^15^^,^^16^ Facilities subject to HIPAA (45 CFR 164.528(a)(1)) are termed “covered entities” (CEs).^15^^,^^17^ To comply with federal policy, a CE follows strict parameters for accessing, storing, or releasing PHI. Data access is limited to those with a need to know.^15^^,^^18^ A CE may include PHI in its analyses for internal QI purposes. However, the CE is not permitted to release PHI to external parties unless explicitly released by a patient (or legal representative), authorized under specific exceptions outlined in HIPAA (45 CFR § 164.512), or deidentified per HIPAA standards prior to any disclosure.^15^^,^^19^

Objectively reporting flexible, patient-centric, or operational efforts to adapt care as a QI effort is different from designing and reporting biomedical, behavioral, or clinical research.^12^^,^^20–25^ However, when enrolled students conduct a QI project at their employer’s site (hereinafter, student-employees), patient and data interactions require unique oversight.

Thus, the primary aims of this report are to (1) proactively establish when governance models are necessary for the population of student-employees conducting QI projects and (2) promote interaction and cooperation when addressing ethical and regulatory standards outside of the institutional review board (IRB) to reduce overregulation for student-employee QI projects.

### QI Instruction in Academic Programs

Academic institutions teach QI methodologies within advanced health care degree programs such as Doctor of Nursing Practice (DNP) or Doctor of Pharmacy.^5^^,^^9^ Appropriate design is imperative to robust QI, and students build competencies within academic curricula.^9^^,^^10^^,^^14^ Students direct QI projects under the supervision of a mentor/preceptor to apply classroom education to practice. The experiential learning effort encourages competency building in design, conduct, and translation to real-world improvements within specific health care organizations.^7^^,^^20^^,^^26–28^ Advanced health care degree programs may need to modify their practices to account for risks unique to dually assigned student-employee QI projects beyond research-based considerations alone.

### When Student Employment Affects Academic QI Projects

A student who completes an assignment to fulfill a degree requirement is engaged in an activity independent of their employment. Combining roles introduces duplicative responsibility that must be appropriately managed and disclosed before the onset of a QI study for academic use. When a student-employee’s QI project does not involve the use of PHI, the activity should not require increased HIPAA-aligned protections. For example, a student can design a project using a pre-post survey design to measure employees’ perceptions before and after a training without disclosing patient data. Student QI projects are likely to be considered low risk if patient outcomes are not a part of the activity.^20^^,^^24^^,^^29^

### Completion of QI Projects in the Workplace

Although a *facility* may possess the authority to design and approve practices for its QI purposes, a single employee in their student capacity is unlikely to be the sole arbiter of a data transfer. Elusive legal responsibilities may arise when an individual’s academic progression involves data transfer outside the CE.^15^^,^^27^^,^^30^^,^^31^ The duties of employment grant access to data and systems that an external entity would not typically possess. A student-employee’s activities within a CE could breach the internal privacy protections afforded to institutions, because the work is no longer limited to health care operations per HIPAA but now includes the student-employee’s education.^19^^,^^23^^,^^32^

Student-employees conducting QI should access data in the same way as an external party would if the study design includes PHI. Data transferred outside the CE can be released only under stringent standards or contractual agreements.^16^ Thus, any transmission or storage of identifiable data to an academic program assignment, a student-owned computer, or an outside IT system risks incongruency with disclosure standards and creates an increased probability for a PHI breach. Health care institutions embed HIPAA-aligned privacy in day-to-day work. Yet, an inadvertent transfer of any identifiable PHI to an academic server or IT system could lead to privacy breaches ranging from mild (eg data on age or dates of medical services) to disastrous (eg identifiable data on disease state or treatments).^18^^,^^33^ However, with appropriate resource allocation and collaborative planning, both health care organizations and academic programs can proactively manage risks. Academic institutions can design effective strategies to guide the management of student-employees to prevent PHI breaches and protect patient safety. Although a review process may resemble that of an IRB, the purposes and scope are substantially different.

### Differences in QI Projects and Human Subjects Research

The medical and bioethics literature dating back over 20 years documents the struggles between practitioners and IRBs when definitions used in human subjects research are applied to QI.^15^^,^^34–38^ Ambiguity in the review process increases the risk of overregulation. For the purposes of this article, the term “overregulation” is viewed as IRB review of a QI project when the involvement of human participants does not meet the definition of “Research” outlined within the Common Rule.^11^^,^^35^^,^^37^^,^^39^ Institutions add unnecessary administrative burden when programs require a “just in case” review or when collaborating organizations request documentation to mitigate potential institutional risk or liability. In addition, graduate departments may require confirmation of ethical considerations to prevent academic missteps and fulfill graduation requirements.^24^^,^^38^ The requirement to submit a QI project application to the IRB creates a circular argument: submit a description for review to ensure the IRB does not need to review.^39^ Institutions engaged in academic QI projects should now consider formally updating their processes to reduce the potential for overregulation.^11^^,^^34^ The Common Rule contains distinct categories for participant risks and benefits; QI does not follow the same structure, and institutions should not put irrelevant barriers to nonresearch activities intended to improve patient care.^15^^,^^35^^,^^40^

### When QI Projects Require IRB Review

Although a QI project may address organizational and operational questions about employee behavior or human care, it is rarely framed as a research question.^24^ QI projects do not bring about generalizable change in the same way as human subjects research does.^21^^,^^28^ Often, IRBs use a litmus test centered around the intent to publish to distinguish whether a proposed project is “generalizable,” a formal, yet undefined term within the Common Rule.^23^^,^^29^ In 2015, the second version of Standards of Quality Improvement Reporting Excellence, or SQUIRE 2.0, was formed to provide consensus on QI reporting.^2^ SQUIRE 2.0 defined a similar term “generalizability” as “The likelihood that the intervention(s) in a particular report would produce similar results in other settings, situations, or environments (also referred to as external validity).” When a QI project reaches the likelihood of generalizability, a project team may need to seek additional review from the IRB or its assigned personnel (eg IRB office staff).^25^^,^^35^^,^^40^^,^^41^

### Ambiguity in Oversight Exists Independent of Student Involvement

Concerns about overregulation and role confusion do not hinge on the inclusion of students. Even without student involvement, confusion can arise from subtle differences in interpretation between QI and research projects. Federal regulators and academic institutions with robust QI and research operations emphasize the need to consider QI projects outside of the IRB’s scope.^11^^,^^25^^,^^34^^,^^37^^,^^42^ Despite publicly posted guidance, interpretations of QI projects and human subjects research remain unclear, inconsistent, or subject to debate.^22^^,^^25^^,^^35^^,^^39^

In the past, components of the U.S. Department of Health and Human Services (HHS), including the Office for Human Research Protections (OHRP) and the Centers for Medicare & Medicaid Services, scrutinized the differences between QI and research.^6^^,^^43^ For example, in the mid-2000s, a published article about practices in catheterization procedures garnered federal attention from OHRP because the organization believed that the authors had crossed over from traditional QI to research.^22^^,^^39^^,^^41^^,^^44^ Publication of the article led to investigative inquiries from OHRP. A publicly available determination shows the highly nuanced distinctions debated between the parties. ^41^ Professional medical organizations also joined the discussion about the importance of developing safe patient standards via QI.^22^^,^^39^ As a result, OHRP later released clarifying statements on the separation between the two activity types.^35^^,^^41^

In 2006, the Hastings Center, a nonpartisan biomedical ethics research institute, published an extensive analysis of scenarios and developed recommendations for implementation.^6^ The report states, “We believe that an organization should develop its own approach to bringing the QI activities in this category into conformity with ethical standards.”^6^ However, health care organizations or academic institutions with risk-averse approaches may use past negative public cases to shape current policy and be resistant to change. An institutional mechanism to avoid public scrutiny or legal concerns by confining the review process solely within the IRB contributes to overregulation.

### Is IRB Review Necessary or Sufficient for QI Projects?

Regardless of the IRB’s interpretation, academic projects remain entangled with the requirements between sites of student QI projects and educational programs.^12^^,^^20–23^^,^^45^^,^^46^ Dependence on an IRB as the sole review body opens the possibility of downplaying other legal and systemic requirements. Even when QI projects appear straightforward and aimed at provider education, an IRB’s framework does not inherently require review or approval of workplace policy compliance or state and local laws.^11^^,^^12^^,^^23^^,^^34^^,^^40^^,^^46^ Thus, without a defined framework, institutions may overlook considerations of site access, security, and liability and insufficiently assess the risk involved. Ideally, a designated individual or committee would review all components before the QI project begins.

IRB oversight of PHI release can fulfill an academic institution’s broader regulatory purposes. Institutions may delegate their IRB to serve a dual role as a privacy board governing the authorization for PHI release, waiver, or deidentification requirements.^15^^,^^19^ Beyond an institution, journals that publish work related to QI may require disclosure of the ethical review parameters.^24^ Even when an institution delegates extra duties to the IRB, any factor outside of the IRB’s primary focus on human subjects research protections introduces the risk of overtaxing and bottlenecking an already strained system.^12^^,^^22^^,^^40^^,^^46^ With shrinking budgets in higher education and a strained workforce in the IRB review profession, duplicative work is not an option.^46–48^ Thus, in standard QI projects with no research component, IRB review alone is neither necessary nor sufficient.

### Models for QI Review and IRB Collaboration

Several models proposed over the past 2 decades provide mechanisms for reviewing academic QI projects outside the traditional IRB route.^11^^,^^25^^,^^34^^,^^37^^,^^49^ Importantly, each requires an institutional commitment to delegate the review to a specific body and a programmatic commitment to support the initiative. For example, Szanton et al^37^ developed a partnership between an IRB office and a DNP program. The parameters trigger IRB review only when applicable by using a prereview process through a liaison.^37^ The group observed that the method substantially cut the volume of reviews routed to the IRB during the given observational period.

Similarly, Foote et al^34^ described a system to divert the majority of their DNP QI projects from IRB review requirements. Again, the volume of IRB-required reviews was reduced, this time by over 96% over 2 years.^34^ The results highlight the tremendous amount of review resources that programs can potentially reroute within an institution to prevent overregulation.

In 2022, McIltrot et al^11^ described an innovative strategy adopted by a school of nursing that considers the various responsibilities and levels of risk associated with external sites. This model serves as an additional exemplar for institutions to build a structure for QI project review practices within the institution and to define the IRB’s or other institutional offices’ involvement (eg legal counsel). Expanding these parameters may further address gaps in the data protection of data used in assignments or publications.

Finally, a recent article by Denison et al^12^ introduced a specialized QI review process intended to avoid overregulation and better outline expectations for students. The authors developed a quality improvement review board (QIRB) with distinct policies and staffing to specifically address the volume of QI projects and to provide comprehensive support and guidance. Furthermore, early results from the QIRB model suggest that students’ publication rates improve upon project completion. Although each model differs in its exact structure and implementation, the clear connection is the removal of the majority of QI project review from the IRB. Training designated reviewers to flag the small number of QI studies proposing to apply study procedures with a rigid interventional design or those that affect clinical decisions is a comprehensive method to address the multitude of concerns unique to QI.^36^

### Legal and Regulatory Risk Awareness Unique to Student-Employee QI Projects

In this work, the author draws on the existing literature and regulatory guidance to present a model for addressing risk in cases where student-employees conduct academic QI projects at their workplaces. Institutions can streamline practices to triage most QI projects away from IRB review and elevate only potential risks for studies that cross into a research-based design.^11^^,^^34^^,^^37^ Once identified, the academic institutions and QI sites may want to consider predefined project topics suitable for a student-employee’s degree program. However, if academic programs do not want to limit the topics or structures available to any student conducting a QI project, a risk picture profile ensures that proper considerations are evident.

### When to Consider Governance Models in Student QI Projects

The need for a governance structure appears when one or more scenarios arise during a project. These scenarios are visualized in Figure 1 as a heatmap and described in additional detail in this section, along with cumulative mechanisms for risk mitigation.

1. A student in an academic program with a QI project requirement plans to publish results.
  - Risk: Low. Student presence, assessment, or observation at the QI site must follow the appropriate policies.
  - Conduct training to outline the basics of QI design, HIPAA basics, and awareness of research versus QI triggers.
2. A student proposes a QI project utilizing PHI.
  - Risk: Moderate. CEs must protect against inadvertent PHI transfer to external IT systems. However, transfers are managed in accordance with standard practices (eg training, agreements, patient authorization, or privacy board or IRB waivers), provided that the CE is aware of the activity in advance of any disclosure.
  - In addition to the training described in low-risk scenarios. Build awareness into instruction by ensuring that students seek out the information required to access PHI at the site through official channels (eg privacy officer). The awareness exercise may be graded or preparatory. The exercise intends to familiarize students with resources available at QI sites, providing specific experiential learning.
  - The use of PHI requires data security considerations. Any transfer or use of data is outlined and defined by the site and the student.
3. The project involves a student-employee leading a QI project at their place of employment.
  - Risk: High. Potential for conflicts in physical and digital access granted solely for purposes of employment. There is potential for legal confusion if the academic institution’s IRB is responsible for reviewing PHI outside its facilities. QI sites may be unaware of the student-employee project and any external data transfer.
  - Adding this layer of employment also increases the responsibility for mitigation steps. Following training, resource location, and data security protections, the use of agreements to outline the student-employee’s role helps protect patient safety. Any potential for inadvertent interventional procedures not intended to be QI for the site must be carefully reviewed. Changes to patient care, or PHI crossover, must be avoided unless clear boundaries and oversight are in place. The potential for research activities may also be a time to consult with an IRB to determine whether informed consent practices or related protections apply.

**Figure 1. The risk/governance heatmap tool assists institutional decision-making in student and student-employee QI projects. As student involvement becomes more complex, the need for governance increases. Institutions can manage risks with mitigating actions by layering training, awareness, data security, and agreements.**
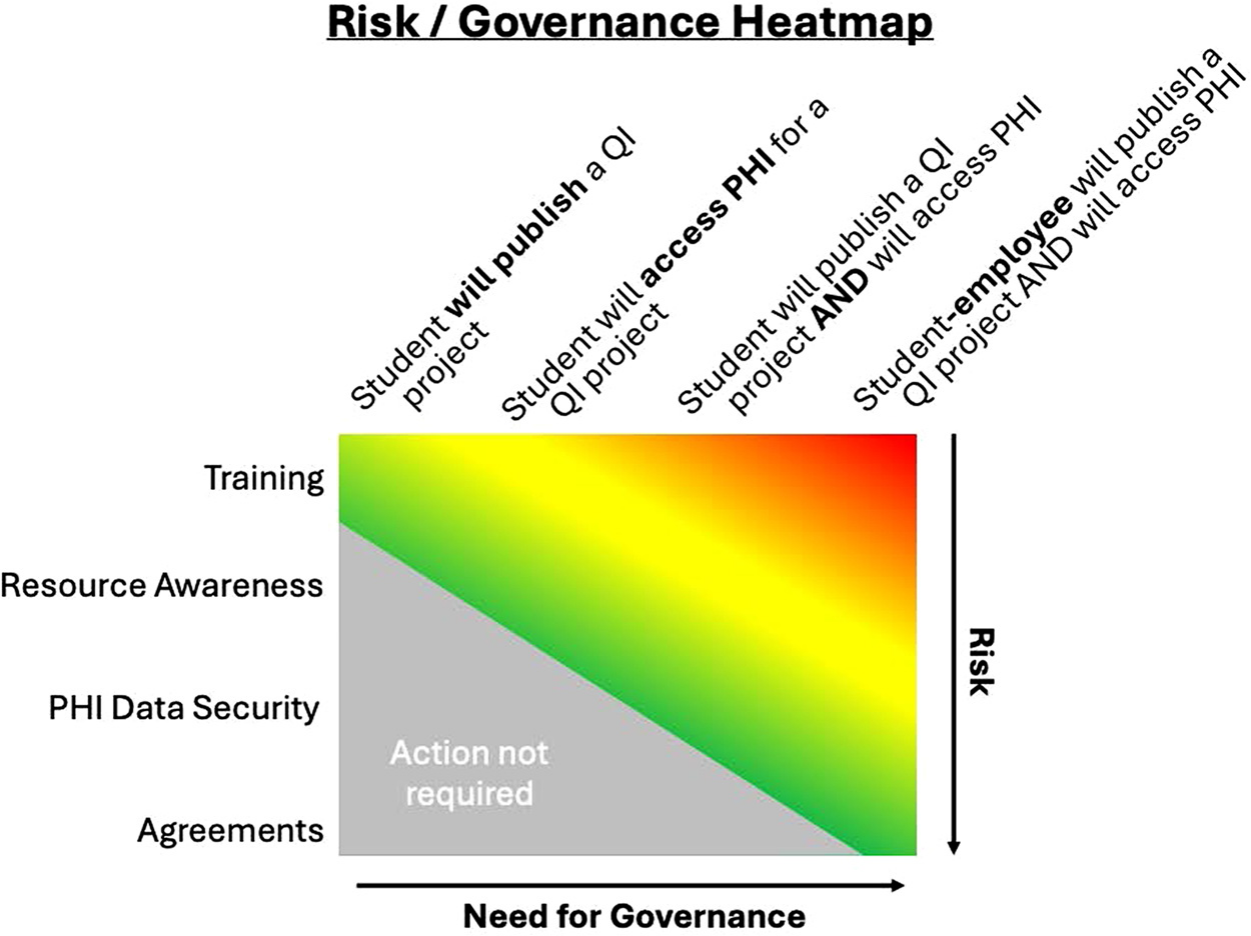


Figure 1 illustrates the increased need for governance structures as the student’s role and relationship to the QI site change.

All parties review and resolve the inherent potential for real or perceived conflicts of interest, data security, and patient privacy, tailored to the activity’s risk level. For example, when students conduct retrospective medical chart reviews as academic QI projects, the activities may detect trends and provide insight into patient outcomes.^1^^,^^6^ However, slight deviations from standard practices may have considerable implications if a student-employee improperly crosses PHI and research publication practices.^15^ Once an institution commits to a need for structured governance, a common framework can be applied across collaborating parties.

## Recommendations

### Model of Shared Responsibilities, 5Circle Student Employee Venn for Quality Improvement

The proposed model is the 5Circle Student Employee Venn for QI (5SEVenn-QI), which uses a 5-part Venn diagram to denote shared responsibilities in student-employee QI projects. Each overlap represents targeted responsibilities and actions that fulfill the shared responsibilities to protect patient data and safety. 5SEVenn-QI displays each institution’s responsibilities, actions, and results but does not prescribe how the parties must operate. The variability of programs and institutions conducting QI mandates flexibility and open discussion to generate effective plans. With the current body of literature on QI review practices, institutions may adapt their own structure from peer institutions.^12^^,^^21^^,^^23–25^^,^^49^^,^^50^

The primary parties in 5SEVenn-QI are the academic institution, the student-employee, and the QI project site. Each operates its programs independently to meet internal and external stakeholder standards.^25^^,^^45^ Both the academic institution and QI project site may also find opportunities for institutional contracts and agreements, such as reliance on a single IRB^29^ or affiliation agreements that establish governance models and definitions for interactions (Figure 2, “Contracts & Agreements”).

**Figure 2. The 5SEVenn-QI model establishes relationships among the parties, shared actions, and expectations for results in student-employee QI projects.**
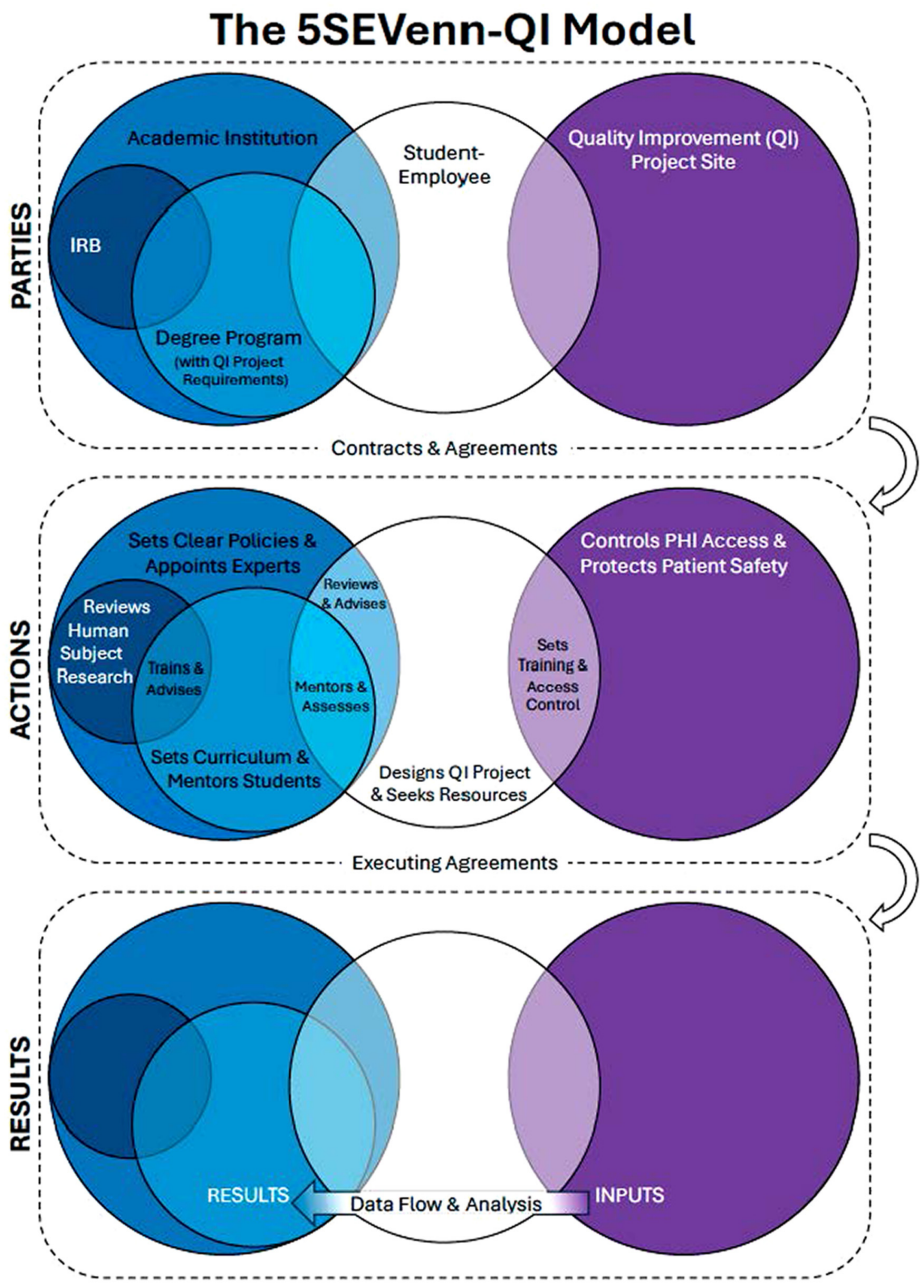


### 5SEVenn-QI Actions

The academic institution must set clear policies for instruction and educational objectives. It administers the degree program (eg the DNP program), sets curricular standards for students, and maintains expertise and personnel among its faculty and preceptors. The academic institution funds and organizes the IRB review processes to fulfill ethical and regulatory requirements involving human subjects research. 5SEVenn-QI charges the IRB only with reviewing, exempting, approving, or denying any applicable human subjects research activities. However, overlap between the IRB and the degree program is necessary to ensure that both are aware of the boundaries and details that distinguish QI activities from research. Prior published models describe a cohesive partnership between the IRB and the degree program.^11^^,^^12^^,^^21^^,^^34^

The QI site is responsible for protecting patients and associated PHI. In addition to training its personnel, it must develop organizational controls aligned with HIPAA and other federal, state, and local laws (Figure 2, “Sets Training & Access Control”). In the 5SEVenn-QI structure, the site is responsible for imparting knowledge and exercising control over the proper use of data, including PHI, and over any limitations on outside use.

A student-employee serves as the nexus for 5SEVenn-QI, linking the academic institution to the QI project site. Student-employees must understand all relationships and reporting responsibilities and maintain a contact point for each interface. Students in educational degree programs may need guidance from the academic institution on any legal, IT, or compliance reviews required for the project (Figure 2, “Reviews and Advises”). The student intends that the QI project results will flow directly back to the degree program as an assignment. Therefore, the data flow relationship must include permissive transfer at the overlaps between the student and the academic institution (Figure 2, “Data Flow & Analysis”). Finally, the degree program must leverage its faculty and preceptors to mentor the student in meaningful project design and in assessment of the student’s understanding (Figure 2, “Mentors and Assesses”).

### When the 5SEVenn-QI Model Is Appropriate

5SEVenn-QI is a decision-making tool for all parties to adopt and revisit regularly, ensuring that all those responsible can proactively anticipate standards in accordance with applicable compliance frameworks. The respective parties should evaluate any process changes or policy standards required to protect patients and their data for all student-employee QI projects. An overlap in the 5SEVenn-QI model means that layered risk mitigation steps can be added proportionately to the activity.

### Training From the IRB to the Academic Program and Student-Employee

Akin to many regulatory and legal programs, it is logical to begin with awareness training. Whenever possible, academic institutions and QI sites must designate compliance responsibilities to experts beyond the IRB, but the IRB does have a role in the 5SEVenn-QI model. Personnel experienced with the inner workings and nuanced regulations related to human subjects research (eg IRB director or IRB member) can provide a standardized lesson on the intent of research to generate generalizable knowledge. Although faculty or preceptors may have experienced the IRB review process, the IRB reviewer’s perspective against the Common Rule, HIPAA Privacy Rule, or institutional requirements is essential for appropriate training delivery.^15^^,^^29^ Advice must be clear, interactive, and consistent across the academic institution. To incentivize expectations, employers could offer student-employees a nominal tuition remission or support for educational supplies for completing training, meeting checkpoints, and reporting results.^3^

### Building Resource Awareness Into Instruction

Next, this model proposes that instructors encourage students to locate key resources from any entities serving as QI study sites (eg their employers) early in project planning. The academic program is responsible for communicating the expectation that student-employees follow site QI practices, even with existing workplace access to data or patients. Facilities follow distinct processes when initiating QI.^11^^,^^34^^,^^51^ Locating resources may be challenging in large or multisite facilities. Thus, QI project development in tandem with onsite resources is crucial for time savings. A site’s policies should outline the expected documentation and systematic supervision required. Any direct interactions with patients must be clear at the beginning of the project.

Institutions and academic programs should proceed cautiously when student QI projects pose the potential to affect patient health (eg sustaining life, hospice, or infection control). Each program and institution has unique quality needs but certainly one size does not fit all. Hospice nurses may operate QI under far different standards than emergency room nurses or retail pharmacists do. Although no universal mold exists, partnerships and training can standardize internal processes across sites. Considering assessment criteria, data access, and distribution of results presents a learning opportunity to establish the most effective mutual benefit for the student and the QI project site.

### Using Agreements to Clarify Responsibilities

If the QI site plans to transfer PHI to a student-employee, then all parties must cooperate to understand the storage processes for all QI projects using identifiable or partially identifiable data. A straightforward agreement between the parties documents acknowledgment of all responsibilities. Presumably, if CEs transfer PHI to an external institution using standard templates and mechanisms, the risk is substantially lowered. Again, delays or bottlenecks could occur if institutions process high volumes of contractual agreements. Thus, institutions can empower designees to develop decision tools and authorize low-risk contracts while continuing to elevate activities with higher risk.

Academic institutions may need to explore options with their legal counsel for business associate agreements, data use agreements, or memoranda of understanding as primary mechanisms to establish uniform expectations for data and system permissions, set project expectations, and govern the sharing of results. Institutions that routinely partner with the same CEs may favor a blanket approach promoting uniform interactions with the QI site. Following the execution of an agreement, the parties can create standard operating procedures for the activity, defining the engagements and who is involved in decision-making in the event of exceptions.

### Frequency of Preventive Actions in the Context of Routine PHI Breaches

Although no published cases reference academic QI project breaches of PHI, examples of inappropriate disclosure were highlighted by Calhoun et al^32^ during academic instruction of physician assistant students. The case studies summarized six real examples of disclosures occurring from students’ confusion during instruction and clinical rotations, which demonstrate how easily breaches occur in conversation or instruction. Likewise, a 2018 review of information about 1138 reported PHI breaches revealed that employees taking PHI home or forwarding it to personal accounts or devices accounted for 6.4% of cases, or 74 instances of breach, thus providing evidence of the continuing need for preventive training to prevent PHI crossover to personal accounts or devices.^52^ Calhoun et al^32^ emphasized that “Students need to understand that whatever happens with patients or is disclosed by patients in the clinical arena must stay within that arena.” This principle is directly relevant to the student-employee role, as even minor, nonlitigated, and likely less detectable risks could still lead to financial, legal, or reputational damage to institutions and patients.

## Discussion

### Implications for Change in QI: Knowing When to Regulate and When Not to Overregulate

In this work, the author provides a background on the convoluted relationships between QI and research activities. Moreover, the interaction with instruction and student employment continues to be complex more than 20 years after initial publication. The model includes a preemptive description of activities that could trigger increased needs for structured governance. The 5SEVenn-QI model broadly assesses when an institution should establish an organized governance structure within its institutional programs. The implications of this change offer significant collaborative potential to develop template agreements and operating procedures that institutions can share to reduce overregulation and to concentrate IRB review efforts on higher-risk studies.

Training must be a regular collaborative effort between all representatives. Instructors clearly state the expectation that if a QI project proposes interventional research for generalizable knowledge, an IRB must review, approve, or exempt the project. The early understanding will help prevent overregulation of QI studies and focus the IRB’s efforts only where necessary. To avoid confusion and delay in student graduation requirements, defined points of contact should be established for the review of any instances involving student access to PHI or involvement in conducting human subjects research.

### Limitations

The author’s interpretation of the available literature forms the basis for the proposed framework. After searches of legal cases, publicly posted PHI breaches, and web searches, very little beyond the aforementioned OHRP correspondence is available for use as an example. Superficially, the lack of information could mean that the author’s interpretation does not adequately consider the robust procedural standards within QI project development. Another possibility is that the lack of cases does not reach a measurable or reportable threshold. Indeed, a 2023 report from the US Government Accountability Office highlighted limitations to OHRP’s bandwidth to conduct audits of IRB practices.^53^ In addition, the US Department of Health & Human Services only requires public reporting of PHI breaches involving 500 or more people, thereby limiting the detectability of less severe or otherwise resolved cases. Future studies could explore the distinction between the lack of cases and visibility through interviews with QI sites or by analyzing available policies on PHI use in QI. Measuring implementation effectiveness is valuable for multiple health care disciplines. However, the majority of published peer-reviewed literature focuses on DNP programs, thereby skewing perspectives on this field.

Furthermore, this model does not address any distinction between participating in a research activity and a mandatory QI activity. The 5SEVenn-QI scope includes only the relationships between student-employees and their academic programs and workplaces. The model does not address a unique issue about employee autonomy in QI projects when outcome measures assess employees’ competency, understanding, or compliance with new standards. A participant-employee must understand any foreseeable repercussions if a supervisor or accreditor has access to the research data from an academic program. IRBs routinely assess risks to reputation or employment in the context of human subjects research and may need to contribute to policies built around this concept. Building a model around the participant-employee role is novel. It may lead to a pathway for further expansion of the heatmap (Figure 1) and the 5SEVenn-QI model’s interface between student-employee and QI site participant-employees for purposes of an academic assignment.

QI projects limited to training exercises or employee surveys do not pose the same risks as those that propose using PHI or patient health outcomes. A blanket process without a gradient-style risk consideration could be a reason why overregulation is so prevalent in the literature.^6^^,^^12^^,^^21–23^^,^^25^^,^^32^^,^^34^^,^^38^^,^^40^^,^^43^^,^^49^ Institutions can now use the heatmapping process to determine the level of risk they are willing to accept and design their governance structures based on the 5SEVenn-QI model. Thus, the model sufficiently addresses the aim of establishing novel risk-proportional guidance for institutions on when to consider a structured governance framework (Figure 1). The 5SEVenn-QI model (Figure 2) addresses the author’s second aim to establish ways to review student-employee QI projects outside the IRB through other institutional bodies responsible for legal and privacy functions beyond the Common Rule. The development of this model highlights specific interactions to consider the perspectives and dimensions of student-employee QI projects.

## Conclusions

QI is an internal process grounded in the health care system and patients’ needs. Institutions are poised to mitigate risk when their QI project-producing academic programs follow transparent policies and governance structures that support modern, effective health care. Academic degree programs teach the methodologies that encourage students to learn QI project design and conduct. Both confusion and overregulation occur in QI projects with or without students and may disincentivize publication and sharing unique QI. After over 20 years of literature describing this concern, the need for stronger models is evident.^6^^,^^12^^,^^43^

Academic programs with QI projects and IRBs can effectively work together to develop simple solutions and define clear paths for appropriate review. By using a standardized approach, institutions can maximize students’ confidence in QI, reduce the administrative burden on already strained academic faculty and IRB offices, and, most importantly, protect and improve patient safety and privacy.

## Disclosures

The author has nothing to disclose.

## Funding

The author received no funding for this research.

CE Quiz
